# Synthesis and Characterization of Bioactive Oligoitaconates with Amino Acid Functional Groups for Tissue Engineering

**DOI:** 10.3390/ijms27010324

**Published:** 2025-12-28

**Authors:** Marta Chrószcz-Porębska, Sylwia Waśkiewicz, Tomasz Gołofit, Agnieszka Gadomska-Gajadhur

**Affiliations:** 1 Faculty of Chemistry, Warsaw University of Technology, Noakowskiego 3 Street, 00-664 Warsaw, Poland; tomasz.golofit@pw.edu.pl; 2 Faculty of Chemistry, Silesian University of Technology, Strzody 9 Street, 44-100 Gliwice, Poland; sylwia.waskiewicz@polsl.pl

**Keywords:** thio-Michael addition, amino acid-polymer adducts, itaconic acid, polymer post-polymerization modification, physicochemical properties

## Abstract

Improving the hydrophilicity and tissue adhesion of polymers remains a significant challenge in tissue engineering and is often addressed by introducing functional groups that enhance polymer–tissue interactions. In this field, L-cysteine (Cys) and N-acetyl-L-cysteine (NAC) are particularly interesting due to their functional carboxyl and amine groups, which are prone to hydrogen bonding. Following this trend, this study (i) investigated the feasibility of grafting Cys or NAC onto the linear oligoitaconates via thio-Michael addition and (ii) examined the influence of amino acid incorporation on the material’s physicochemical properties. NMR-based calculations confirmed nearly 100% addition efficiency for Cys and a slightly lower, but still high, efficiency for NAC. FT-IR spectra confirmed thiol-based addition, as signal from the Cys/NAC S–H stretching vibrations was not observed in the adduct’s spectra. The obtained adducts showed thermal stability up to 200 °C and glass transition temperatures below −20 °C. They were soluble in common organic solvents, except for Cys adducts with oligo(propylene itaconate) and oligo(hexylene itaconate), which were water-soluble only. Due to the low molecular weight (below 1000 g/mol) of oligoitaconates, their adducts cannot serve as standalone scaffold components. However, they showed potential for use as modifiers for high-molecular-weight polylactide or poly(ɛ-caprolactone)-based scaffolds.

## 1. Introduction

Itaconic acid (IA) is an unsaturated dicarboxylic acid. It was discovered in 1836 as a by-product of citric acid distillation [1]. Subsequently, it was found to be biosynthesized in vivo by the fungal species *Aspergillus*, *Ustilago*, and *Candida* through glycolysis and the tricarboxylic acid cycle [2,3,4]. In 2011, it was reported that it can be synthesized by mammalian immune cells [5].

The chemical structure of IA is similar to that of methacrylic acid. Both of them contain a carbon–carbon double bond that facilitates radical polymerization and a carboxylic group that enables functionalization or crosslinking [6]. However, IA’s structure is slightly more complex. Its additional carboxylic group makes IA more versatile for chemical modification, hydrophilic, and biodegradable. These features and renewable production position itaconic compounds as a sustainable and biocompatible alternative to methacrylic compounds in the medical field, particularly in biomaterial applications [7,8,9]. Itaconic compounds have a wide range of applications in pharmaceuticals, biomaterials, and medical devices [10,11,12,13,14,15,16,17,18,19,20]. IA and its derivatives are utilized as drug delivery systems due to their polymerization capabilities and functional groups [16,17,18,19,20] and as antimicrobial agents [12,13,14,15]. Itaconic compounds also significantly impact mammalian metabolism, particularly in immune cells such as macrophages. This renders them valuable for treating inflammatory conditions, including rheumatoid arthritis, inflammatory bowel disease, and neuroinflammatory disorders [21,22].

Itaconic compounds are gaining prominence in tissue engineering as a basis for creating scaffolds [23,24,25,26]. Their non-toxicity to living cells, natural degradability into non-harmful by-products, antibacterial activity, chemical versatility, and tunable mechanical properties make them ideal for such applications. However, there is a growing interest in improving the tissue adhesion of itaconic compounds, as their intrinsic adhesion to tissues is relatively limited compared to other adhesive materials. This limitation arises from the lack of strong tissue-interactive groups.

Enhancing polymer tissue adhesion often involves the introduction of functional groups or structures that promote stronger molecular interaction with tissues. Those modifications include (i) introducing catechol groups [27,28,29], (ii) incorporating thiol, hydroxyl, or carboxyl functional groups [30,31], (iii) grafting hydrophilic side chains [32], and (iv) introducing bioactive molecules such as amino acids and peptides [33,34,35,36]. Among the latter, grafting amino acids, particularly L-cysteine, has gained increasing attention [37].

L-cysteine (Cys) conjugation to polymers has been shown to enhance tissue adhesion, improve hydrophilicity, and prolong the degradation time of polymers. This is attributed to the unique chemical structure of Cys, which is the only natural amino acid containing a thiol group [38]. Introducing Cys into polymers enhances tissue adhesion through covalent and non-covalent interactions. The thiol group in the Cys molecule can form stable covalent disulfide bonds with thiol-containing subdomains in proteins. At the same time, non-covalent interactions involve the formation of hydrogen bonds between the Cys amine or carboxylic groups and functional groups in tissue proteins. These interactions are crucial in cell adhesion by facilitating molecular contact, improving adhesion stability, and contributing to cohesive interactions between the polymer and tissues [37,39].

Itaconic compounds show potential for modification through a Michael addition reaction, in which their unsaturated double bonds act as Michael acceptors. However, there are only a few studies that describe the modification of polyitaconates through this approach. Several papers describes the functionalization of linear poly(itaconates), including poly(butylene itaconate) modified with hexamethylenediamine [40], poly(octyl itaconate) modified with diethylamine [41,42], poly(dodecyl itaconate) and poly(icosyl itaconate) modified with N-methylbenzyl amine and diallyl amine [43], via aza-Michael additions that required harsh reaction conditions, such as elevated temperatures [40,41,42,43], the use of catalysts [41,42,43], toxic organic solvents [43], and prolonged reaction times [40,41,42,43]. Moreover, these modifications often suffered from low addition efficiencies [40,41,42,43] or undesired intramolecular cyclization of the polyester backbone [40], which significantly limited their applicability. Thio-Michael routes for modifying linear poly(itaconates) have rarely been reported. The examples involving poly(dodecyl itaconate) and poly(icosyl itaconate) functionalized with thiophenol, 2-mercaptoethanol, thioglycerol, or cystine methyl ester also required the use of catalysts and toxic solvents and showed limited efficiency [43].

The objective of this study was to (i) synthesize polymer-amino acid adducts through an environmentally friendly, catalyst-free approach that avoids the use of auxiliary reagents and toxic solvents, generating no harmful by-products; (ii) examine how amino acid incorporation influences the physicochemical properties of the resulting materials, including degree of addition (*AD*), thermal stability (*Td*), glass transition temperature (*Tg*), and solubility; and (iii) assess the potential of the obtained materials for use in tissue engineering to manufacture scaffolds via non-solvent induced phase separation (NIPS) and electrospinning methods.

**Figure 1:**
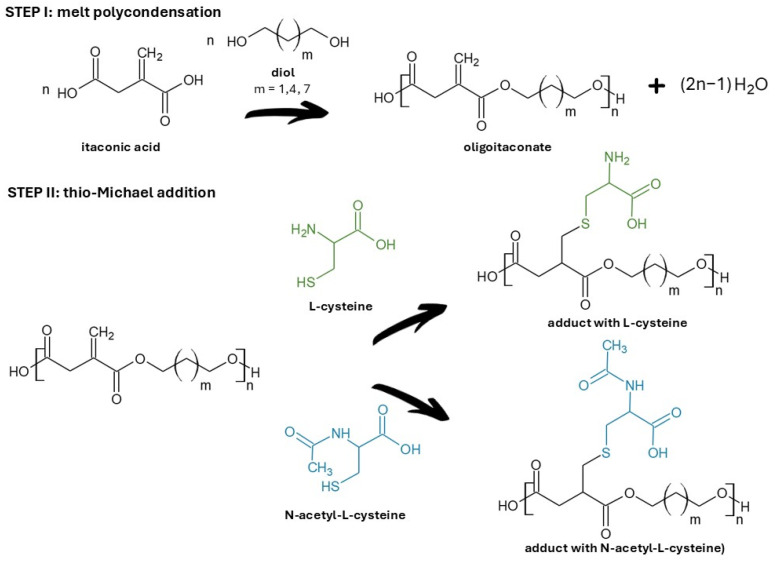
Synthesis of oligoitaconate adducts with L-cysteine and N-acetyl-L-cysteine. The green color indicates the L-cysteine molecule, while the blue color indicates the N-acetyl-L-cysteine molecule.

Linear oligoitaconates (Figure 1) were chosen as the polymer backbone due to their reactive double bonds, which enable efficient grafting of functional molecules. Although the applied oligoitaconates exhibit relatively low molecular weights, they remain attractive for biomedical applications because their chemical structure ensures biodegradability and non-toxicity to living cells. The applied oligoitaconates varied in the length of the diol segment, ranging from three to nine methylene (-CH_2_-) moieties, in order to assess whether it influences the thio-Michael addition reaction or the physicochemical properties of the obtained adducts. Cys and NAC (Figure 1) were used because their thiol groups readily undergo thio-Michael addition with the oligoester double bonds, leaving their carboxyl and amino groups free to form hydrogen bonding, enhancing hydrophilicity and adhesion to biological tissues. This approach will allow us to obtain novel biodegradable and non-toxic oligoesters with enhanced hydrophilicity. In addition to the development of new oligoitaconate–Cys/NAC adducts, our study introduces a versatile method that overcomes the issues related to the material’s hydrophobicity, which can be extended to other polymers containing reactive unsaturated bonds.

In this pilot study, we focused on the synthesis and physicochemical characterization of adducts to identify materials suitable for further research on cell scaffolds manufactured via NIPS and electrospinning methods. Since (i) the length of the diol segment in polyesters is known to influence scaffold properties, such as degradation rate, hydrophilicity, mechanical performance, and crystallinity, and (ii) the obtained adducts varied in diol segment length, future studies are planned to investigate the relationship between the adducts’ diol segment length and the resulting scaffold properties. Future work will also involve assessing the cytotoxicity and cell proliferation of the modified scaffolds.

## 2. Results

**Figure 2:**
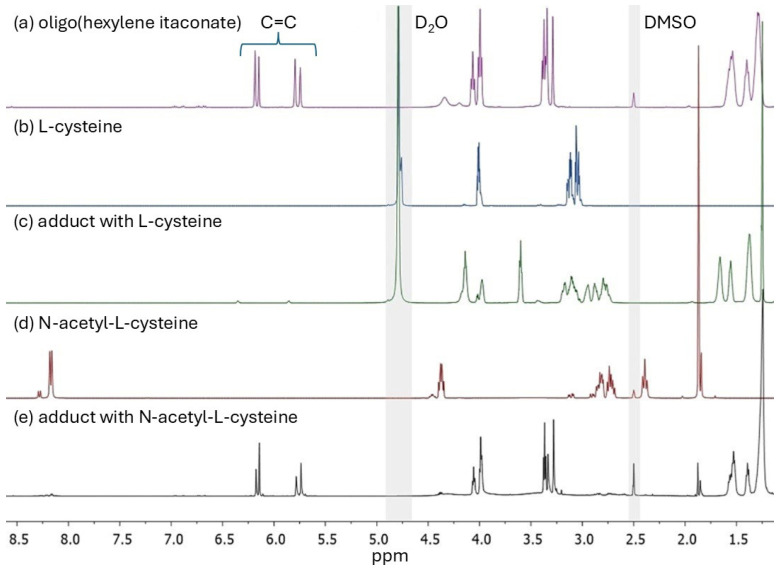
^1^H NMR spectra of (**a**) oligo(hexylene itaconate), (**b**) L-cysteine, (**c**) Michael’s adduct with L-cysteine, (**d**) N-acetyl-L-cysteine, and (**e**) Michael’s adduct with N-acetyl-L-cysteine. The gray highlights indicate signals from the deuterated solvents.

**Figure 3:**
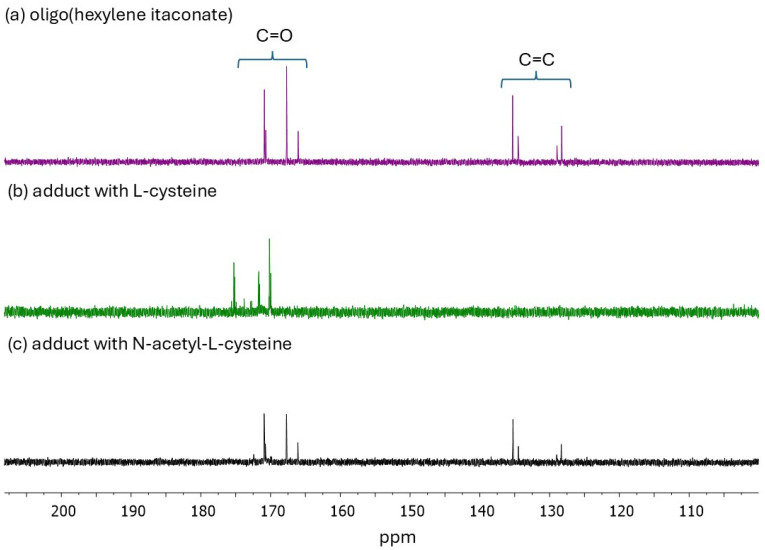
^13^C NMR spectra of (**a**) oligo(hexylene itaconate), (**b**) Michael’s adduct with L-cysteine, and (**c**) Michael’s adduct with N-acetyl-L-cysteine.

**Figure 4:**
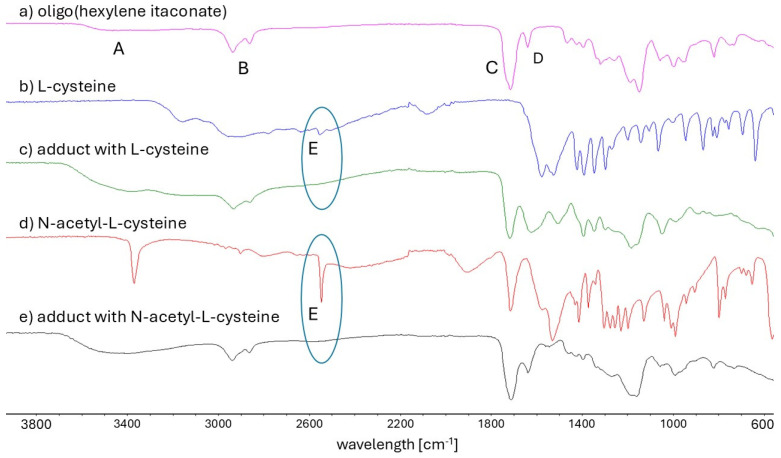
FT-IR spectra of (**a**) oligo(hexylene itaconate), (**b**) L-cysteine, (**c**) Michael’s adduct with L-cysteine, (**d**) N-acetyl-L-cysteine, and (**e**) Michael’s adduct with N-acetyl-L-cysteine. A sign corresponds to a signal from the stretching vibrations of hydroxyl groups derived from the oligoester -CH_2_OH end group. B sign corresponds to a stretching vibrations of methylene (-CH_2_-) groups in the diol segment. C sign corresponds to a stretching vibrations of the carbonyl group. D sign corresponds to stretching vibrations of the double bonds. E sign corresponds to stretching vibrations of the thiol (-SH) group. The blue circle highlights the disappearance of the thiol group signal in the adduct spectra.

In this study, three oligoesters of itaconic acid and linear diols were synthesized and subsequently subjected to thio-Michael addition with L-cysteine (Cys) and N-acetyl-L-cysteine (NAC). Figure 1 shows the chemical structures of the obtained oligoitaconates and their thio-Michael adducts. The chemical structures of the obtained oligoesters were confirmed by ^1^H NMR (Figure 2), ^13^C NMR (Figure 3), and FT-IR (Figure 4) spectroscopy.

The ^1^H NMR spectrum of oligo(hexylene itaconate) (OHI), presented in Figure 2a, represents the spectra of synthesized oligoitaconates. The signals observed in the 5.75–5.80 ppm and 6.15–6.18 ppm ranges correspond to the double bonds in the oligoitaconate segment. Peaks at 3.99 ppm, 3.35 ppm, and within 1.2–1.7 ppm are attributed to the -C(O)-O-CH_2_, -O-CH_2_, and -CH_2_- groups of the diol segment, respectively, while the signal at 3.29 ppm corresponds to the -CH_2_- group of the itaconic acid segment. The ^1^H NMR spectra also indicate the occurrence of side reactions during polycondensation (Figure S1). Low-intensity signals at 3.12 ppm, 3.26 ppm, and within the range of 3.45–3.9 ppm correspond to protons involved in the Ordelt reaction, which occurs between the itaconic acid double bond and the diol hydroxyl groups. Peaks from 6.62 to 6.92 ppm are associated with the mesaconic acid and itaconic anhydride isomerization reaction. Furthermore, signals in the 1.8–2.4 ppm range suggest the free radical polymerization of itaconic acid double bonds. The ^1^H NMR spectra of the Michael adducts (Figure 2c,e) reveal a significant reduction in double bond signal intensity, approaching 100% in the case of the Cys adduct. Additionally, the spectra of the NAC-based Michael adducts show no detectable signal from the thiol group, which would have appeared at 2.4 ppm, as observed in the NAC spectrum.

^13^C NMR spectra of OHI further confirmed the presence of double bonds in the oligoester structure (Figure 3a), with corresponding peaks observed within 127–135 ppm. The ^13^C NMR spectra of thio-Michael adducts (Figure 3b,c) confirmed the occurrence of Michael addition. Peaks corresponding to the double bonds initially observed within 120–140 ppm were not observed in the Cys adduct spectrum and exhibited reduced intensity in the NAC adduct spectrum.

The FT-IR spectrum of OHI, presented in Figure 4a, serves as a representative spectrum of the synthesized oligoitaconates. The FT-IR spectra confirm the presence of characteristic functional groups within the oligoitaconate structure. A broad peak (A) observed within the 3650–3400 cm^−1^ range corresponds to the stretching vibrations of hydroxyl groups derived from the oligoester -CH_2_OH end group. Sharp signals at 2934 cm^−1^ and 2860 cm^−1^ (B) are attributed to the stretching vibrations of methylene (-CH_2_-) groups in the diol segment. Additionally, peaks at 1713 cm^−1^ and 1637 cm^−1^ correspond to the stretching vibrations of the carbonyl group (C) and double bonds (D), respectively. The FT-IR spectra of the Michael adducts (Figure 4c,e) revealed the disappearance of the signal associated with the stretching vibrations of thiol (-SH) groups (E), initially observed at 2551 cm^−1^ in both the Cys (Figure 4b) and NAC (Figure 4d) spectra.

Before modification, the oligoitaconates were analyzed for their acid number (*AN*), ester number (*EN*), esterification degree (*ED*), and molecular weight (*MW*). The results are summarized in Table 1.

The *AN* and *EN* were determined using the titration method. The *AN* values ranged from 122 to 193 mg KOH/g and decreased with an increasing number of methylene groups in the diol segment. OHI and oligo(nonylene itaconate) (ONI) showed similar *AN* values (*p* > 0.05), whereas oligo(propylene itaconate) (OPI) showed higher values (*p* ≤ 0.05). A similar trend was observed for *EN*, with values ranging from 691 to 448 mg KOH/g. The *ED* values of oligoitaconates were similar (*p* > 0.05), averaging 86%. The *MW* of oligoitaconates ranged between 562 and 960 g/mol and increased with the lengthening of the diol segment. The polydispersity index (*Đ*) of oligomer molecular weights ranged from 1.50 to 1.84. OPI and OHI exhibited similar *Đ*, whereas ONI showed a higher *Đ* value.

**Figure 5:**
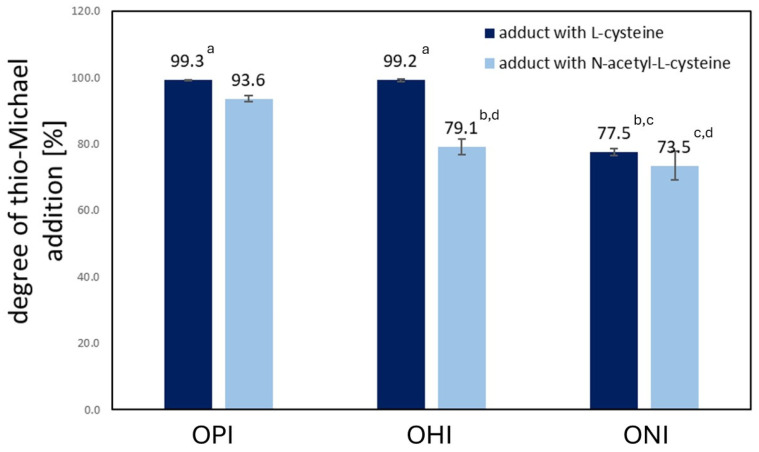
The degree of thio-Michael addition (*AD*) of L-cysteine and N-acetyl-L-cysteine to oligoitaconates. Lowercase letters indicate statistically insignificant differences (*p* > 0.05) with a column (Kruskal–Wallis with Mann–Whitney U post hoc test).

Further, the addition degree (*AD*) was calculated by comparing the double bond content in oligoitaconates and their thio-Michael addition products (Table S2). As shown in Figure 5, the *AD* ranged from 77.7 to 99.3% when Cys was used. These values were higher than those obtained with NAC, where the *AD* values ranged from 73.8 to 93.5%. In both cases, the *AD* decreased as the length of the diol used for oligoitaconate synthesis increased. OPIA1 and OHIA1 showed the highest and similar (*p* > 0.05) *AD* values, averaging 99.25%. OHIA1 showed the lowest *AD* among adducts with Cys, which were similar (*p* ≤ 0.05) to those obtained for OHIA2 and ONIA2.

**Figure 6:**
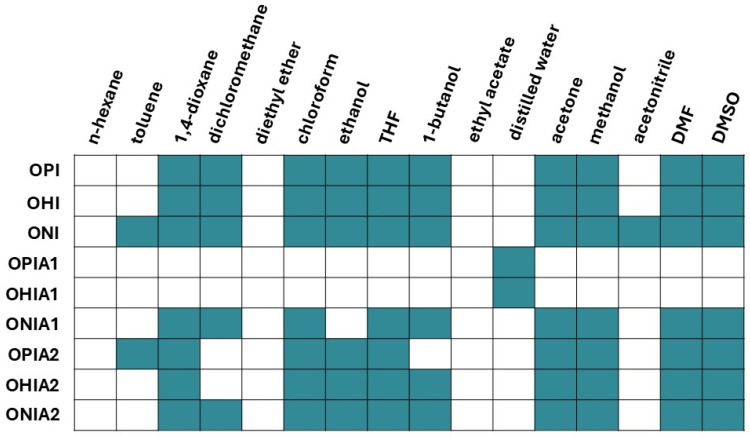
Solubility of oligoitaconates and their adducts in organic solvents. The green color indicates that the compound is soluble in the solvent, while the white color indicates insolubility.

The obtained oligoitaconates and thio-Michael adducts were tested for their thermal properties and solubility. Those results are summarized in Table 2 and Figure 6.

Table 2 shows the results of differential scanning calorimetry (DSC) and thermogravimetry (TG) analysis. The representative thermograms of the studied materials are presented in the Supplementary Materials (Figures S2 and S3). As can be seen, the glass transition temperature (*Tg*) of oligoitaconates ranged from—53 to −38 °C and decreased with the increasing diol segment length. Functionalization via the Michael addition reaction led to an increase in *Tg* values. Adducts with Cys showed *Tg* within the range of −55 and −21 °C, whereas the NAC adducts showed *Tg* values between −44 and −25 °C. Thermogravimetric analysis (TG) revealed that oligoitaconates exhibited high thermal stability, with a mass loss of 30% observed within the temperature range of 263 to 331 °C. It was also observed that the thermal stability of oligoesters increased as the diol segment length increased, as all the studied decomposition temperatures increased with the lengthening of the oligomer repeating unit. Functionalization via the Michael addition reaction led to a significant decrease in *Td_5%_*. As shown in Table 2, its value ranged from 70 to 125 °C for Cys adducts and from 73 to 134 °C for NAC adducts. However, the *Td_30%_* and *Td_50%_* values remained high and ranged between 204–246 and 244–285 °C for Cys adducts and between 305–351 and 331–371 °C for NAC adducts.

Figure 6 presents the results of the solubility tests. Before modification, oligoitaconates exhibited solubility in most organic solvents except n-hexane, toluene, diethyl ether, ethyl acetate, distilled water, and acetonitrile. Following modification with Cys, OPIA1 and OHIA1 remained soluble only in distilled water, while ONIA1 lost its solubility in toluene, ethanol, and acetonitrile. In contrast, adducts with NAC retained their solubility in the majority of tested solvents, except for dichloromethane, in which OPIA2 and OHIA2 were insoluble, and 1-butanol, in which OHIA2 exhibited insolubility.

## 3. Discussion

Improving tissue adhesion of polymeric materials is essential for their effective use in biomedical applications such as wound healing, drug delivery systems, and tissue engineering scaffolds, the latter being of particular importance. Enhanced tissue adhesion promotes cellular attachment and growth, facilitates tissue integration, prevents inflammatory responses, and ensures the structural integrity of the tissue. To enhance tissue adhesion, polymers are chemically functionalized. The unique chemical structure of L-cysteine (Cys) makes it especially valuable for polymer modification, as it can form both covalent and non-covalent interactions with tissue proteins.

Following this trend, we developed oligomer–amino acid adducts by grafting cysteine (Cys) and N-acetyl-L-cysteine (NAC) onto linear oligoitaconates with different diol segment lengths via Michael addition (Figure 1). These novel materials are designed for the fabrication of tissue-engineering scaffolds using electrospinning and non-solvent-induced phase separation (NIPS) techniques. To our knowledge, such adducts have not been reported previously. As this study represents an initial investigation, we focused on the synthesis and evaluation of the fundamental physicochemical properties of the obtained adducts to explore their potential for use in tissue engineering scaffold manufacturing.

Three esters of itaconic acid with 1,3-propanediol (PDO), 1,6-hexanediol (HDO), and 1,9-nonanediol (NDO) were successfully synthesized via solvent-free melt polycondensation as confirmed by ^1^H NMR (Figure 2), ^13^C NMR (Figure 3), and FT-IR analyses (Figure 4). In this process, the carboxyl (-COOH) groups of itaconic acid reacted with the hydroxyl (-OH) groups of diols, forming ester bonds (-COO-). During the reaction, water molecules were generated as by-products and removed under reduced pressure. The resulting products were straw-colored, viscous resins.

Synthesized oligoitaconates exhibited a low acid number (*AN*) and a high ester number (*EN*), indicating a high degree of esterification (*ED*). This was further confirmed by ^1^H NMR analysis, which showed that *ED* values exceeded 85% (Table 1). The oligoitaconates were characterized by relatively low molecular weights (*MW*, *MW* < 1000 g/mol) with a polydispersity index ranging from 1.5 to 1.85, typical for mass polycondensation. This suggests that the synthesized polymer chains consisted of approximately four repeating units. Consequently, these low-*MW* oligoitaconates are unsuitable as standalone components of tissue engineering scaffolds, as short polymer chains would not provide sufficient chain entanglement [44,45], which is crucial in the electrospinning process and leads to uneven pore distribution in NIPS-manufactured scaffolds. Therefore, it will be necessary to facilitate their application by incorporating a carrier polymer, such as polylactide or poly (ɛ-caprolactone). Such a solution is recognized in the literature [46,47,48].

The Michael addition reaction to the double bonds of oligoitaconates was carried out using Cys and NAC. In this process, the α,β-unsaturated double bonds of oligoitaconates functioned as Michael acceptors, while the thiol (-SH) group of the amino acid molecules served as Michael donors (nucleophiles). As a result, Cys and NAC were covalently grafted onto the oligomer backbone via Michael addition, forming stable carbon–sulfur bonds. The remaining free carboxylic (-COOH) and amine (-NH_2_, -NH(COCH_3_)) groups of amino acids are capable of forming hydrogen bonds, contributing to enhanced hydrophilicity and improved adhesion to biological tissues [49,50].

The applied Michael addition reaction was characterized by its simplicity. The reaction was carried out in a water-acetone mixture at room temperature, without a catalyst, and for 24 h. A detailed analysis of the ^1^H NMR (Figure 2) and FT-IR (Figure 4) spectra revealed that the addition of Cys and NAC occurred through the thiol group. No signal corresponding to the thiol group (expected at around 2.4 ppm) was detected in the ^1^H NMR spectrum of NAC adducts, and the FT-IR spectra of both Cys and NAC adducts showed a complete disappearance of the signal at 2551 cm^−1^, associated with the thiol group’s stretching vibrations.

Michael’s addition was characterized by high efficiency (Figure 5), with the most significant addition observed for Cys, achieving nearly 100%. For NAC, the highest addition degree (*AD*) reached 94%. A correlation was found in both cases, suggesting that the length of the diol segment in oligoitaconates affects the *AD*. This can be explained by the increasing flexibility of the polymer chain, which could obscure double bonds, thereby limiting their accessibility to the functional groups of Cys and NAC. These observations were further supported by DSC analysis (Table 2), which showed a decrease in the glass transition temperature (*Tg*) of the oligoitaconates with increasing diol segment length. As reported in the literature, the lower the *Tg*, the higher the mobility of the polymer chain [51,52]. A similar concept, regarding the hindered access of Cys/NAC functional groups to the double bonds of the oligoester, can be applied to explain the lower *AD* of NAC. The acyl group in NAC likely creates steric hindrance, impeding the addition process. Additionally, the observed decrease in the *AD* values with increasing diol length may be related to the decreasing hydrophilicity of the oligoesters [53]. Since the addition reaction was carried out in an acetone/water mixture, oligoesters synthesized using longer diols may undergo chain aggregation, further restricting access to the double bonds.

To maximize the *AD*, a 4-fold molar excess of Cys and NAC was used relative to the number of double bonds in the oligoester. However, due to the significant excess of Cys/NAC used in the reaction, a purification step was necessary to remove unreacted compounds from the crude product, as unreacted Cys or NAC in high concentrations could adversely affect biological tissues [54,55] and influence the physicochemical properties of the obtained adducts. The purification process utilizes the differences in solubility between the adducts and unreacted Cys/NAC in water. Most thio-Michael adducts were insoluble in water, except for OPIA1 and OHIA1. However, their dissolution rate was slower than that of free Cys, allowing for the effective separation and purification of the final products. The effectiveness of the developed purification method for removing unreacted Cys/NAC was validated through ^1^H NMR (Figure 2) and FT-IR analysis (Figure 4). In both Cys and NAC adducts FT-IR spectra, no characteristic signals associated with the stretching vibrations of the thiol group were observed, which would have indicated the presence of unreacted residues. Additionally, no signal from the NAC thiol group was detected on the ^1^H NMR spectra of NAC adducts. These results suggest that any potential residual amounts of Cys or NAC, if present in adducts, were below the detection limits of the applied analytical techniques.

Purified adducts were evaluated for their thermal properties (Table 2) and solubility (Figure 6) to assess their potential in scaffold fabrication via electrospinning and NIPS methods.

Thermal analysis included the determination of the decomposition temperature (*Td*) and the glass transition temperature (*Tg*). The *Tg* was evaluated to determine whether the adducts would be in a glassy state at human body temperature. The *Td* was determined to assess the thermal stability of the adducts and to define the maximum temperature at which the adducts can be safely processed.

The functionalization of oligoitaconates with Cys and NAC increased *Tg*, indicating a decrease in polymer chain mobility. This can be attributed to a greater steric hindrance in the grafted oligomer chain, which restricts the relaxation of the polymer chains. Such behavior is known in the literature [56,57,58]. Despite this increase, the *Tg* values of the obtained adducts were lower than −20 °C, indicating sufficient chain mobility to facilitate continuous fiber formation, essential in the electrospinning process. As the obtained adducts are intended for manufacturing materials for use in the human body, their *Tg* values below the human body temperature (37 °C) are beneficial, as they ensure the stability of the materials’ physicochemical and mechanical properties.

The unmodified oligoitaconates exhibited high thermal stability, with a 50 mass loss observed at temperatures above 350 °C. Compared to the unmodified oligoitaconates, Michael adducts showed slightly lower thermal stability. This is likely due to the incorporation of thermally less stable Cys and NAC groups, undergoing decomposition around 200 °C [59,60]. The modified oligoitaconates showed a lower Td_5*%*_, which might suggest faster initial degradation. However, those results are likely not associated with the reduction in thermal stability of the adducts, but rather with the residual traces of water from the purification step. As all adducts remained stable up to 200 °C, they showed potential for use in scaffold manufacturing and post-processing treatment without inducing material degradation.

The solubility of Michael’s adducts was assessed as a key parameter concerning manufacturing scaffolds via NIPS and electrospinning methods. Since the obtained adducts cannot be used as standalone components of tissue engineering scaffolds, but rather as modifiers for scaffolds based on high molecular weight polymers like polylactide or poly(ɛ-caprolactone), they must be soluble in the same organic solvents as these polymers. The obtained results confirm the suitability of the adducts for scaffold fabrication (Figure 6). All Michael’s adducts with NAC demonstrated solubility in common organic solvents such as dichloromethane, chloroform, THF, acetone, and DMF, while their counterparts with Cys exhibited limited solubility. Like pure Cys, the adducts of OPI and OHI with Cys (OPIA1 and OHIA1, respectively) showed solubility only in distilled water. This behavior can be attributed to the synergistic effect of two factors: the *AD* and the hydrophilicity of the diol segments. OPIA1 and OHIA1 were characterized by the highest *AD*, indicating a higher concentration of hydrophilic Cys in the polymer chain compared to the other adducts. Additionally, these adducts were synthesized using the shortest diols—PDO and HDO. Therefore, it can be concluded that the high concentration of hydrophilic Cys in the oligomer chain overcomes the hydrophobic nature of the oligoitaconate chain exerted by the diol segment, which is reflected in the water solubility of the adducts. In contrast, the ONI adduct with Cys, which had a 20% lower *AD*, was insoluble in water due to its lower Cys content and the more hydrophobic NDO segment.

## 4. Materials and Methods

### 4.1. Materials

Acetone (POCH, Gliwice, Poland), acetonitrile (POCH, Gliwice, Poland), 1-butanol (POCH, Gliwice, Poland), chloroform (Chempur, Piekary Śląskie, Poland), deuterated dimethyl sulfoxide (Deutero GmbH, Kastellaun, Germany), deuterium oxide (Deutero GmbH, Kastellaun, Germany), dimethyl sulfoxide (DMSO, Chempur, Piekary Śląskie, Poland), dichloromethane (HPLC grade, Sigma Aldrich, St. Louis, MO, USA), dichloromethane (POCH, Gliwice, Poland), diethyl ether (Chempur, Piekary Śląskie, Poland), dimethylformamide (DMF, POCH, Gliwice, Poland), 1,4-dioxane (POCH, Gliwice, Poland), ethanol (POCH, Gliwice, Poland), ethyl acetate (POCH, Gliwice, Poland), n-hexane (POCH, Gliwice, Poland), 1,6-hexanediol (Thermo Fisher Scientific, Waltham, MA, USA), hydrochloric acid (HCl, 1 M, Chempur, Piekary Śląskie, Poland), itaconic acid (Sigma Aldrich, St. Louis, MO, USA), L-cysteine (Merck KGaA, Darmstadt, Germany), methanol (Chempur, Piekary Śląskie, Poland), N-acetyl-L-cysteine (Chemat, Gdańsk, Poland), 1,9-nonanediol (Thermo Fisher Scientific, Waltham, MA, USA), 1,3-propanediol (Thermo Fisher Scientific, Waltham, MA, USA), sodium hydroxide (NaOH, 1 M, Chempur, Piekary Śląskie, Poland), tert-butanol (t-BuOH, Sigma Aldrich, St. Louis, MO, USA), tetrahydrofuran (THF, POCH, Gliwice, Poland), toluene (Chempur, Piekary Śląskie, Poland), were used as received

### 4.2. Oligoester Synthesis

Oligo(propylene itaconate) (OPI), oligo(hexylene itaconate) (OHI), and oligo(nonylene itaconate) (ONI) were synthesized according to the following procedure. Itaconic acid (0.23 mol, 30.00 g) and diol (0.23 mol, from 17.51 to 36.86 g, depending on the alkyl chain length (Table S1)) were introduced into a 100 mL round-bottom flask. A combined hot-plate magnetic stirrer device heated the reaction mixture via an oil bath for 6 h. The oil bath temperature was set to 140 °C. The polycondensation by-product (water) was removed from the reaction system under reduced pressure. The obtained product was in the form of a liquid resin.

The optimization of process parameters for polycondensation was achieved experimentally by varying synthesis conditions, including reaction temperature and time. The presented parameters represent the conditions that enable the synthesis of an ungelled polyester with a high degree of esterification (conversion of carboxylic groups to ester groups) and a low extent of side reactions.

### 4.3. Thio-Michael Addition

L-cysteine (Cys) and N-acetyl-L-cysteine (NAC) were used for the thio-Michael addition.

50 wt.% solution of oligoitaconate (15 g) in acetone was placed in a 250 mL glass beaker. Then, a 10 wt.% water solution of Cys/NAC was added dropwise. Cys/NAC was used in a 4-fold molar excess in relation to the number of double bonds in oligoitaconate (Table S2). The reaction mixture was stirred mechanically at 200 rpm for 24 h at room temperature. The solvents (acetone and water) were removed using a rotary evaporator under a pressure of 30 mbar. The crude product, a mixture of thio-Michael adduct and unreacted Cys/NAC, was agitated three times with 100 mL of distilled water to remove unreacted Cys/NAC. After each shaking step, the mixture was centrifuged for 30 min at 6000 rpm to separate the undissolved polymer from the water. The water was then removed by decantation, and a fresh portion of clean water was added. After being purified, adducts were dried at 60 °C for 24 h. The final product was in the form of a viscous, straw-colored resin.

### 4.4. Nuclear Magnetic Resonance (NMR)

^1^H and ^13^C NMR spectra of oligoitaconates and thio-Michael addition products were recorded in DMSO solutions, with 32 and 256 scans, respectively, utilizing a 400 MHz spectrometer (Agilent, Santa Clara, CA, USA). The samples were prepared by dissolving about 120 mg of the tested polymer in 1 mL of DMSO. An internal standard, tert-butanol (t-BuOH) of known mass, was added to determine the double bond content. The obtained spectra were analyzed using MestReNova (version 6.0.205457).

Based on the ^1^H NMR spectra, the degree of esterification (*ED*) and double bond content in 100 g of oligomer (*x_DB_*) was calculated.

The *ED* was calculated according to the following equation:

(1) $$ED\;\left(\%\right)=\frac{\sum \int DB}{\int Mes+\sum \int DB+\sum \int IA+\sum \int Or+\int rp}\times 100,$$

where

∑∫DB—summarized value of the integral signals from double bond protons of oligoitaconate,
∫Mes—the integral value of the signal from mesaconic acid,
∑∫IA—summarized value of the integral signals from the double bond protons of the unreacted itaconic acid,
∑∫Or—summarized value of the integral signals from the Ordelts reaction,
∫rp—the integral value of the signal from the itaconic acid radical polymerization.

The *x_DB_* was calculated according to the following equation:

(2) xDBmolC=C100g=nt−BuOH9∗∑∫DB2∫t−BuOH9×2×100m,

where

$n_{t-BuOH}$—number of moles of t-BuOH used for NMR analysis (mol),
∑∫DB—summarized value of the integrals from double bond protons,
∫t−BuOH—the value of the integral of the signal from the CH_3_- group of t-BuOH,
m—mass of oligomer sample (g).

Based on the double bond content in 100 g of oligoester before (x_*DB*1_) and after (x_*DB*2_) addition, the addition degree (*AD*) was calculated using the following equation:

(3) $$AD\;\left(\%\right)=\frac{x_{DB1}-x_{DB2}}{x_{DB1}}\times 100,$$

where

$x_{DB1}$—double bond content in 100 g of oligoester before modification (mol_C=C_/100 g),
$x_{DB2}$—double bond content in 100 g of oligoester after modification (mol_C=C_/100 g).

### 4.5. Fourier Transform Infrared Spectroscopy (FT-IR)

FT-IR spectra were recorded using 32 scans within the 4000–400 cm^−1^ range, with the ALPHA spectrometer (Bruker, Berlin, Germany) and the Attenuated Total Reflectance (ATR) technique.

### 4.6. Gel Permeation Chromatography/Size Exclusion Chromatography (GPC/SEC)

The molecular weights (*MW*) and polydispersity index (*PDI*) were analyzed using size exclusion chromatography (SEC) using the Agilent 1260 Infinity (Santa Clara, CA, USA) apparatus. The system was equipped with an isocratic pump, an autosampler, a degasser, a column with a thermostat, and a differential refractometer (MDS RI Detector). Data were collected and processed with Addon Rev. (version B.01.02, Agilent Technologies, Santa Clara, CA, USA) software. *MWs* were determined through calibration with linear polystyrene standards (580–128 900 g/mol). The separation process utilized a pre-column guard (3 μm, 50 × 7.5 mm) alongside two columns: PLgel MIXED-D (5 μm, 300 × 7.5 mm) and PLgel MIXED-E (3 μm, 300 × 7.5 mm). Measurements were conducted at 30 °C in dichloromethane (HPLC grade) as the mobile phase, with a flow rate of 0.8 mL/min.

### 4.7. Acid Number (AN)

A 0.5–1.0 g oligoester sample was dissolved in 25 mL of methanol. Five drops of thymol blue indicator were then added to the solution. The mixture was titrated with 1 M aqueous NaOH solution. The endpoint was indicated by the change in color of the mixture from yellow to blue.

The acid number (*AN)* was calculated according to the following equation:

(4) AN mgKOHg=V−V0×MNaOH×56.1m,

where

V—the volume of 1 M NaOH solution used for sample titration (cm^3^),
$V_{0}$—the volume of 1 M NaOH solution used for blank titration (cm^3^),
$M_{NaOH}$—the concentration of the titration solution (1 M),
m—mass of oligomer sample (g).

### 4.8. Ester Number (EN)

An oligoester sample weighing 0.2–0.5 g was dissolved in 15 mL of methanol and 20 mL of 1 M aqueous NaOH solution and then refluxed for 1 h. After being cooled to room temperature, five droplets of phenolphthalein indicator were added, and the excess NaOH was titrated with 1 M aqueous HCl. The discoloration of the mixture indicated the endpoint.

The ester number (*EN*) was calculated according to the following equation:

(5) EN mgKOHg=V−V0×MHCl×56.1m−AN,

where

V—the volume of 1 M HCl solution used for sample titration (cm^3^),
$V_{0}$—the volume of 1 M HCl solution used for blank titration (cm^3^),
$M_{HCl}$—the concentration of the titration solution (1 M),
m—mass of oligomer sample (g),
AN—acid number (mgKOH/g).

### 4.9. Esterification Degree (ED)

The esterification degree (*ED*) was calculated based on the *AN* and *EN* results, according to the following equation:

(6) $$ED\;\left(\%\right)=\frac{EN}{EN+AN}\times 100,$$

where

AN—acid number (mgKOH/g).
EN—ester number (mgKOH/g).

### 4.10. Differential Scanning Calorimetry (DSC)

DSC measurements were performed in the temperature range of −90 to 100 °C, with a heating rate of 10 K/min, at nitrogen flow (50 mL/min), utilizing the Q2000 DSC analyzer (TA Instruments, Eschborn, Germany). All measurements were performed in standard aluminum crucibles (10 mg sample weight).

DSC thermograms were analyzed using the TA Instrument Universal Analysis 2000 software. The glass transition temperature (*Tg*) was taken as the midpoint of the transition region.

### 4.11. Thermogravimetry (TG)

TG measurements were performed in the 20 to 500 °C temperature range, with a 10 K/min heating rate, at nitrogen flow (100 mL/min) utilizing the SDT Q600 analyzer (TA Instruments, Eschborn, Germany). Analyses were performed on samples weighing between 8 and 12 mg.

The sample weight loss was analyzed using TA Instruments Universal Analysis 2000 software.

### 4.12. Solubility

The solubility of the oligoesters and their adducts was determined for the following solvents: n-hexane, toluene, 1,4-dioxane, dichloromethane, diethyl ether, chloroform, ethanol, THF, 1-butanol, ethyl acetate, distilled water, acetone, methanol, acetonitrile, DMF, and DMSO. A sample weighing 0.1 g was placed into 2 mL glass vials. Then, 1 g of solvent was added. After 24 h of shaking using a vibrating shaker (Heidolph 545-10000-00, Schwabach, Germany), the solubility was determined by visual assessment.

### 4.13. Statistical Analysis

Statistical analysis was performed using Statistica 13.1 software (TIBCO Software Inc., Palo Alto, CA, USA). The results were expressed as average values of five measurements, along with corresponding standard deviations (*SD*), except for the molecular weight and thermal properties, for which the measurements were repeated once. The non-parametric Kruskal–Wallis test with the Mann–Whitney U post hoc test was used to determine the statistical significance of the results, with a significance level (*p*) of 0.05.

## 5. Conclusions

In this study, three linear oligoesters of itaconic acid with diol segments of three, six, and nine methylene moieties (-CH_2_-) were synthesized by melt polycondensation and subsequently subjected to thio-Michael addition reaction with L-cysteine (Cys) and N-acetyl-L-cysteine (NAC). This allowed us to obtain six Michael adducts expected to increase tissue adhesion due to the functional amine (-NH_2_) and carboxyl (-COOH) groups’ ability to form hydrogen bonds with tissue protein.

The applied synthesis procedure requires the following steps: (i) mass polycondensation of itaconic acid and linear diols with a 1:1 ratio, (ii) thio-Michael addition of Cys/NAC to the oligoester double bonds with a 4-fold molar excess of Cys/NAC concerning the number of double bonds in the oligoester, and (iii) purification of crude adduct from the unreacted Cys/NAC. Despite the multistep character of the applied synthesis procedure, it was simple and environmentally friendly, as it did not involve the use of a catalyst or toxic solvents and did not produce any harmful by-products.

The synthesized oligoesters had low molecular weight, meaning the polymer chain comprised four repeating units. This suggests that their adducts could not be applied as a standalone component of scaffolds, as they will not provide sufficient structural integrity. However, they can be used as a modifier of scaffolds based on polylactide (PLA) or poly(ɛ-caprolactone) (PCL). Adducts based on NAC were characterized by (i) solubility in organic solvents, in which PLA and PCL showed solubility, (ii) high thermal stability up to 200 °C, and (iii) low glass transition temperature. This means they will withstand the conditions of scaffold manufacturing and post-processing treatment, highlighting their potential use as tissue engineering scaffold components. Despite the high thermal stability and low glass transition temperature of Cys-based adducts, their use is limited due to their insolubility in water. Therefore, among the three obtained Cys adducts, only ONIA1 can be considered for use in scaffold manufacturing.

## Figures and Tables

**Table 1:** Acid number (*AN*), ester number (*EN*), esterification degree (*ED*), molecular weight (*MW*), and polydispersity index (*Đ*) of oligoitaconates. Lowercase letters indicate statistically insignificant differences (*p* > 0.05) with a column Kruskal–Wallis with Mann–Whitney U post hoc test.

Sample	*AN* (mgKOH/g)		*EN* (mgKOH/g)		*ED* (%)		*MW*	
	avg	*SD*	avg	*SD*	avg	*SD*	*MW* (g/mol)	*Đ*
oligo(propylene itaconate) OPI	193	12	691	42	88 ^a,b^	3	562	1.50
oligo(hexylene itaconate) OHI	129 ^a^	21	509	39	85 ^a,c^	3	690	1.51
oligo(nonylene itaconate) ONI	122 ^a^	11	448	20	88 ^b,c^	2	960	1.84

**Table 2:** Decomposition temperatures (*Td*) and glass transition (*Tg*) temperatures of oligoitaconates and thio-Michael adducts.

Sample	*Td* (°C)				*Tg* (°C)
	*Td_5%_*	*Td_30%_*	*Td_50%_*	*Td_85%_*	
OPI	182	263	360	420	−38
OHI	170	283	381	422	−53
ONI	214	331	396	427	−50
OPIA1	98	204	310	433	−21
OHIA1	125	205	305	401	−22
ONIA1	70	246	351	415	−55
OPIA2	73	244	331	418	−25
OHIA2	76	261	357	418	−31
ONIA2	134	285	371	418	−44

## Data Availability

The raw data supporting the conclusions of this article will be made available by the authors on request.

## Supplementary Materials

The following supporting information can be downloaded at: https://www.mdpi.com/article/10.3390/ijms27010324/s1.

## Abbreviations

The following abbreviations are used in this manuscript:

*A1*	Adduct with L-cysteine
*A2*	Adduct with N-acetyl-L-cysteine
*AD*	Addition degree
*AN*	Acid number
*Cys*	L-cysteine
*DSC*	Differential Scanning Calorimetry
*DMF*	Dimethylformamide
*DMSO*	Dimethyl sulfoxide
*ED*	Esterification degree
*EN*	Ester number
*FT-IR*	Fourier Transform Infrared Spectroscopy
*GPC*	Gas Permeation Chromatography
*HDO*	1,6-hexanediol
*IA*	Itaconic acid
*MW*	Molecular weight
*NAC*	N-acetyl-L-cysteine
*NDO*	1,9-nonanediol
*NIPS*	non-solvent-induced phase separation
*NMR*	Nuclear magnetic resonance
*PDO*	1,3-propanediol
*OHI*	oligo(hexylene itaconate)
*PNI*	oligo(nonylene itaconate)
*OPI*	oligo(propylene itaconate)
*SEC*	Size Exclusion Chromatography
*t-BuOH*	tert-butanol
*Td*	Decomposition temperature
*TG*	Thermogravimetry
*THF*	Tetrahydrofuran
*Tg*	Glass transition temperature
*Đ*	polydispersity
*x_DB_*	double bond content in 100 g of polymer
