# Supplementary material for: Synthesis and Characterization of Bioactive Oligoitaconates with Amino Acid Functional Groups for Tissue Engineering
Source: Int J Mol Sci. 2025 Dec 28;27(1):324. doi: 10.3390/ijms27010324 (PMC12785526; doi:10.3390/ijms27010324)
Supplement: Supplementary file 1 [file ijms-27-00324-s001.zip › ijms-4036355-supplementary.pdf]

Supplementary Materials

Synthesis and Characterization of Bioactive Oligoitaconates with Amino Acid Functional Groups for Tissue Engineering

Marta Chrószcz-Porębska [1,*], Sylwia Waśkiewicz [2], Tomasz Gołofit [1] and Agnieszka Gadomska-Gajadhur [1,*]

[1] Faculty of Chemistry, Warsaw University of Technology, Noakowskiego 3 Street, 00-664 Warsaw, Poland; tomasz.golofit@pw.edu.pl
[2] Faculty of Chemistry, Silesian University of Technology, Strzody 9 Street, 44-100 Gliwice, Poland; sylwia.waskiewicz@polsl.pl
* Correspondence: marta.porebska@pw.edu.pl (M.C.-P.); agnieszka.gajadhur@pw.edu.pl (A.G.-G.)

Table S1. L-cysteine and N-acetyl-L-cysteine moles and masses used for thio-Michael addition.

thio-Michael adduct	L-cysteine		N-acetyl-L-cysteine	
	**mole**	**mass (g)**	**mole**	**mass (g)**
PPIA1	0.079	9.63	-	-
PHIA1	0.059	7.15	-	-
PNIA1	0.052	6.36	-	-
PPIA2	-	-	0.079	16.20
PHIA2	-	-	0.059	12.04
PNIA2	-	-	0.052	10.70

Table S2. The number of double bonds in oligoesters before (x_{DB1}) and after (x_{DB2}) thio-Michael addition.

sample name	x_{DB1} [mol/100g]		x_{DB2} [mol/100g]	
	**avg.**	***SD***	**avg.**	***SD***
PPI	0.135[a]	0.003	-	-
PHI	0.096	0.003	-	-
PNI	0.087[a]	0.002	-	-
PPIA1	-	-	0.0009[a,b]	0.0002
PHIA1	-	-	0.0008[c]	0.0001
PNIA1	-	-	0.0195[a]	0.0023
PPIA2	-	-	0.0086	0.0012
PHIA2	-	-	0.0200	0.0023
PNIA2	-	-	0.0229[b,c]	0.0021

*Lowercase letters indicate statistically significant differences ($p < 0.05$) with a column (Kruskal-Wallis with Mann-Whitney U post-hoc test).

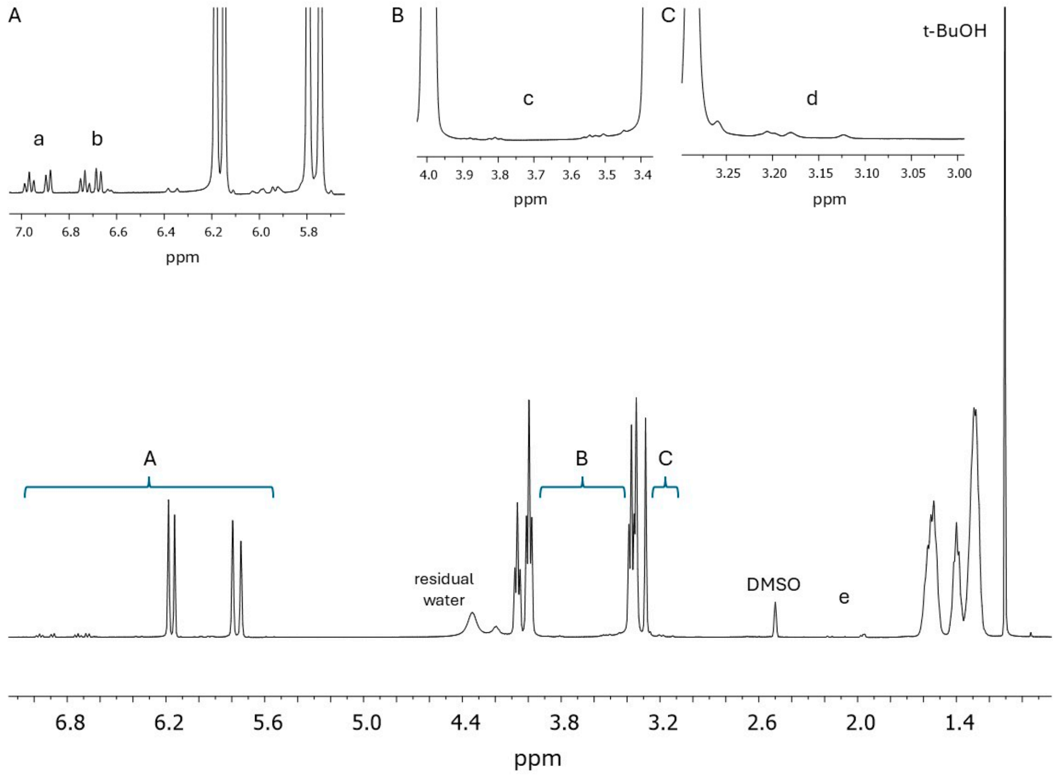

Figure S1. The ^{1}H NMR spectra of oligo(hexylene itaconate) with marked signals of side reactions: a) itaconic anhydride formation, b) isomerization to mesaconic acid, c,d) Ordelt reaction, e) free radical polymerization.

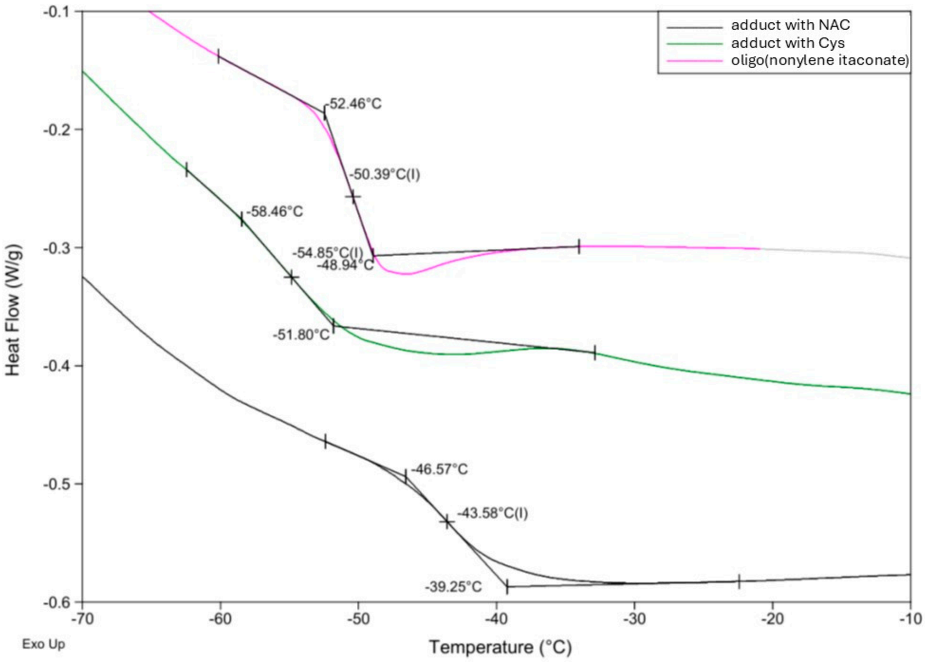

Figure S2. The DSC thermograms of oligo(nonylene itaconate) and its adducts with Cys and NAC showed as representative thermograms of studied materials.

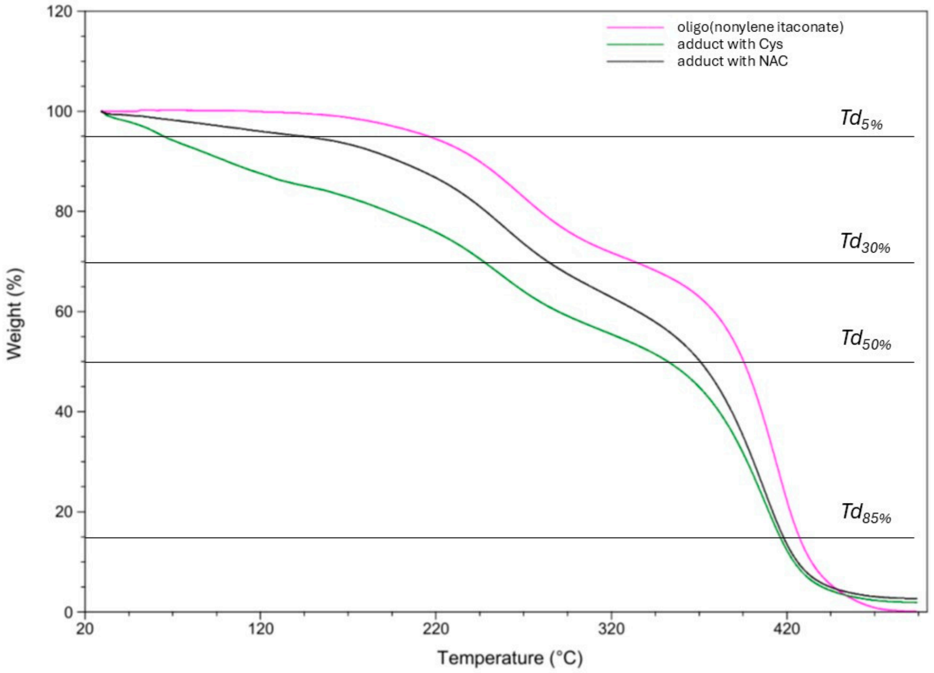

Figure S3. The TG thermograms of oligo(nonylene itaconate) and its adducts with Cys and NAC showed as representative thermograms of studied materials.
